# Use of diffusive gradients in thin-film technique to predict the mobility and transfer of nutrients and toxic elements from agricultural soil to crops—an overview of recent studies

**DOI:** 10.1007/s11356-024-33602-5

**Published:** 2024-05-13

**Authors:** Marin Senila, Eniko Kovacs

**Affiliations:** INCDO INOE 2000, Research Institute for Analytical Instrumentation, Donath 67, 400293 Cluj-Napoca, Romania

**Keywords:** Agricultural soil, Diffusive gradients in thin films, Soil-plant transfer, Nutrient uptake, Heavy metals

## Abstract

The purpose of this review was to survey the recent applications of the diffusive gradients in thin films (DGT) technique in the assessment of mobility and bioavailability of nutrients and potentially toxic elements (PTEs) in agricultural soil. Many studies compared the capabilities of the DGT technique with those of classical soil chemical extractants used in single or sequential procedures to predict nutrients and PTE bioavailability to crops. In most of the published works, the DGT technique was reported to be superior to the conventional chemical extraction and fractionation methods in obtaining significant correlations with the metals and metalloids accumulated in crops. In the domain of nutrient bioavailability assessment, DGT-based studies focused mainly on phosphorous and selenium labile fraction measurement, but potassium, manganese, and nitrogen were also studied using the DGT tool. Different DGT configurations are reported, using binding and diffusive layers specific for certain analytes (Hg, P, and Se) or gels with wider applicability, such as Chelex-based binding gels for metal cations and ferrihydrite-based hydrogels for oxyanions. Overall, the literature demonstrates that the DGT technique is relevant for the evaluation of metal and nutrient bioavailability to crops, due to its capacity to mimic the plant root uptake process, which justifies future improvement efforts.

## Introduction

With the growing of the world’s population, the demand for food is continuously increasing; thus, the agricultural sector goes to intensive farming. This entails the continuous cultivation of crops on soil using a substantial application of fertilizers and pesticides (Chen et al. 2023). Besides the benefits, this may have a negative effect on the environment, due to both the release of the nutrients added in excess and to the soil contamination with potentially toxic elements (PTEs) (Kuziemska et al. 2023). In addition, the development of industrial activities resulted in the release of PTEs in all environmental compartments, including the agricultural soil (Park et al. 2023). PTEs may have extremely harmful effects on the ecosystem, affecting the plant growth and being transferred to the food chain, causing human health risks (Dippong et al. 2022; Zulkernain et al. 2023; Jiang et al. 2023). There are many PTE species that were found in the soil in increased concentrations (Roba et al. 2016; Petrean et al. 2023). Some of the PTEs, like Fe, Zn, Co, Cu, Cr, B, and Mn, are also known as micronutrients for crops, small amounts of these elements being required for plant development. At low concentrations, these PTEs can help some cellular functions in plants, as well as pigment biosynthesis, enzyme activities, photosynthesis, sugar metabolism, respiration, nitrogen fixation, and other functions, but even these elements become toxic at increased concentrations, affecting plant growth (Rashid et al. 2023). Other PTEs such as Cd, Pb, As, Hg, and Sb are non-essential and highly toxic even at low concentrations and affect biota, and finally human health (Briffa et al. 2020; Senila et al. 2022; Thalassinos et al. 2023).

Consequently, the prediction of the nutrient and PTE transfer from soil to the crops has become an increasing concern worldwide (Ning et al. 2021; Gao et al. 2022a, b; Zhou et al. 2023). The key process in the prediction of nutrient and PTE transfer through the food chain is the assessment of their availability in soil for improving the fertilization efficacy, while reducing the soil contamination (Wenzel et al. 2022), but also for preventing the food web contamination through the transfer to crops above the safe limits. Thus, assessing the bioavailability of PTEs and nutrients in agricultural soils has critical implications for food security and for a sustainable agriculture.

Many studies on nutrients and PTEs in soil were based on the measurement of their total concentration, even though their available concentrations would have been more informative. Moreover, in general, the studies on nutrient and PTE availability in soil were carried out by using sequential or single-chemical extraction procedures, but these offer only records of diverse fractions of elements in the soil (Senila 2014; Gao et al. 2022a, b). The diffusive gradients in thin film (DGT) technique was well documented as being a superior tool for the evaluation of metal and nutrient bioavailability to biota due to its capacity to mimic the uptake of elements by the plant roots (Tandy et al. 2011; Zhang and Davison 2015; Marrugo-Madrid et al. 2021). When deployed into the soil, the DGT device introduces a sink for free ions in the soil solution, generating a diffusive flux of those ions into the DGT probe. As the free ions are gradually uptaken by the DGT device, their concentration in the adjacent soil solution decreases causing a disequilibrium among the free ions in the soil solution, their complexes, and their forms fixed onto soil solid phases. In response to the removal of the free ions, labile complexes dissociate, and their forms bound to the soil solid phase can also desorb depending on their availability, being thus resupplied to the soil solution. This uptake mechanism is similar to the plant root uptake into their rhizosphere (Davison and Zhang 2012; Letho 2016). Generally, DGT measurements are mostly independent of the soil characteristics, which could make DGT a good practice for advancing soil quality standards (Tian et al. 2018).

Since several previous review papers (Zhang and Davison 2015; Santner et al. 2015; Marrugo-Madrid et al. 2021; Wei et al. 2022; Guan et al. 2022) and the book *Diffusive gradients in thin-films for environmental measurements* (Davison 2016) presented the concept and theory of DGT, it was the aim of this review to provide an update of the existing DGT knowledge for scientists and practitioners, related to the application of this technique to the agricultural field, for assessing the nutrient and PTE bioavailability in soil. Although the principles of DGT technique were not significantly changed since it was first reported until today, this review aims to provide new trends and findings from its application, or new binding gels developed for various analytes. Since the DGT was found to be generally superior to other techniques in predicting element transfer to crops, this review brings together the newest advancements of the DGT uses on agricultural soil emphasizing the necessity and importance of ensuring food security. The literature published in the last 5 years was mainly analyzed to observe the trend, but several previously published reference works for the DGT development were also considered.

## Methodology to collect data

The search keyword “DGT agricultural soil” was used for the Web of Science Core Collection database, and *n* = 276 documents were found, of which 274 articles (including 3 proceeding papers and 2 review articles) and 2 book chapters. The 143 articles published between 2019 and 2023 were selected and were examined to involve agricultural soil and/or crops. Those dealing with the use of the DGT technique for assessing the bioavailability of metals and nutrients from agricultural soil were included in this literature review.

## DGT applicability for element availability in soil, DGT procedure, and deployment in soil

### Theory of DGT measurement for nutrients/PTEs in soil

**Fig. 1:**
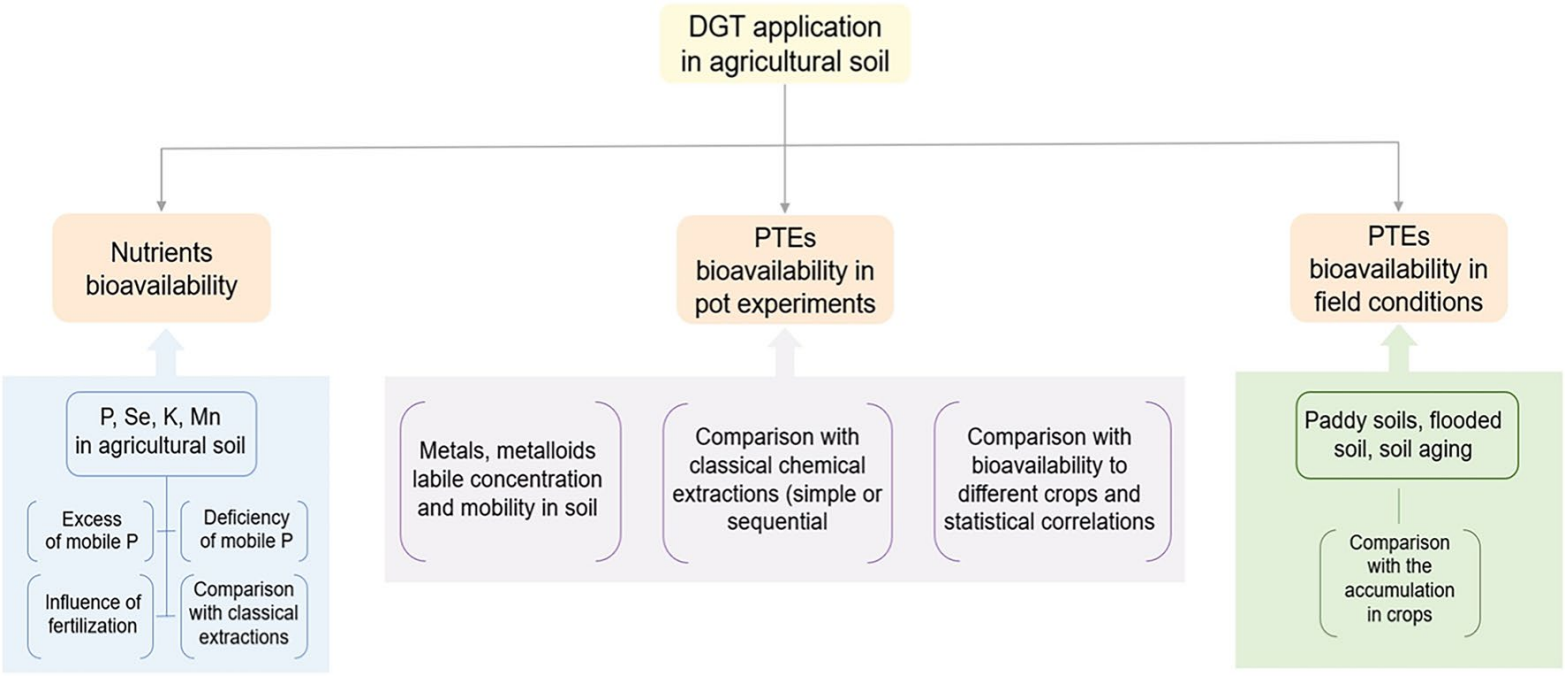
Overview of the DGT applicability in agricultural soil

An overview of the applicability of DGT in agricultural soil is presented in Fig. 1. The DGT technique was created in 1994 by Hao Zhang and William Davison for the determination of trace metals in water (Davison and Zhang 1994). The functioning principle of the DGT technique was widely presented by Davison and Zhang (2016). Here, we are briefly presenting the procedure used for estimating the fractions from soils which are bioavailable to crops.

The DGT technique is founded on Fick’s first law of diffusion, which relates the diffusive flux of analyte to the gradient of the concentration. This flux is controlled by a diffusive layer with a known thickness (*Δg*) which is placed between the analyzed soil solution and a resin gel that accumulates the analytes (Zhang and Davison 1995). The analyte concentration in the analyzed soil solution can be calculated using Eq. (1) (Zhang and Davison 1995):

1 $${C}_{DGT}=\frac{M\times \Delta g}{D\times A\times t}$$

where *M* is the mass of metal bound by the resin gel, *Δg* is the diffusive layer thickness (sum of the diffusive gel and filter paper thickness), *t* is the time for deployment (in seconds), *D* is the diffusion coefficient through the diffusion layer, and *A* is the exposed area to the soil solution.

The mass of analyte accumulated on the resin gel may be eluted from the gel using an appropriate volume of eluents (*V*_*e*_). The measured concentration of the analyte in the eluent (*C*_*e*_), the elution factor (*f*_*e*_), and the volume of the resin gel (*V*_*g*_) are used to calculate *M* using Eq. (2):

2 $$M={C}_e\frac{\left({V}_g+{V}_e\right)}{f_e}$$

### DGT procedure and deployment in soil

**Fig. 2:**
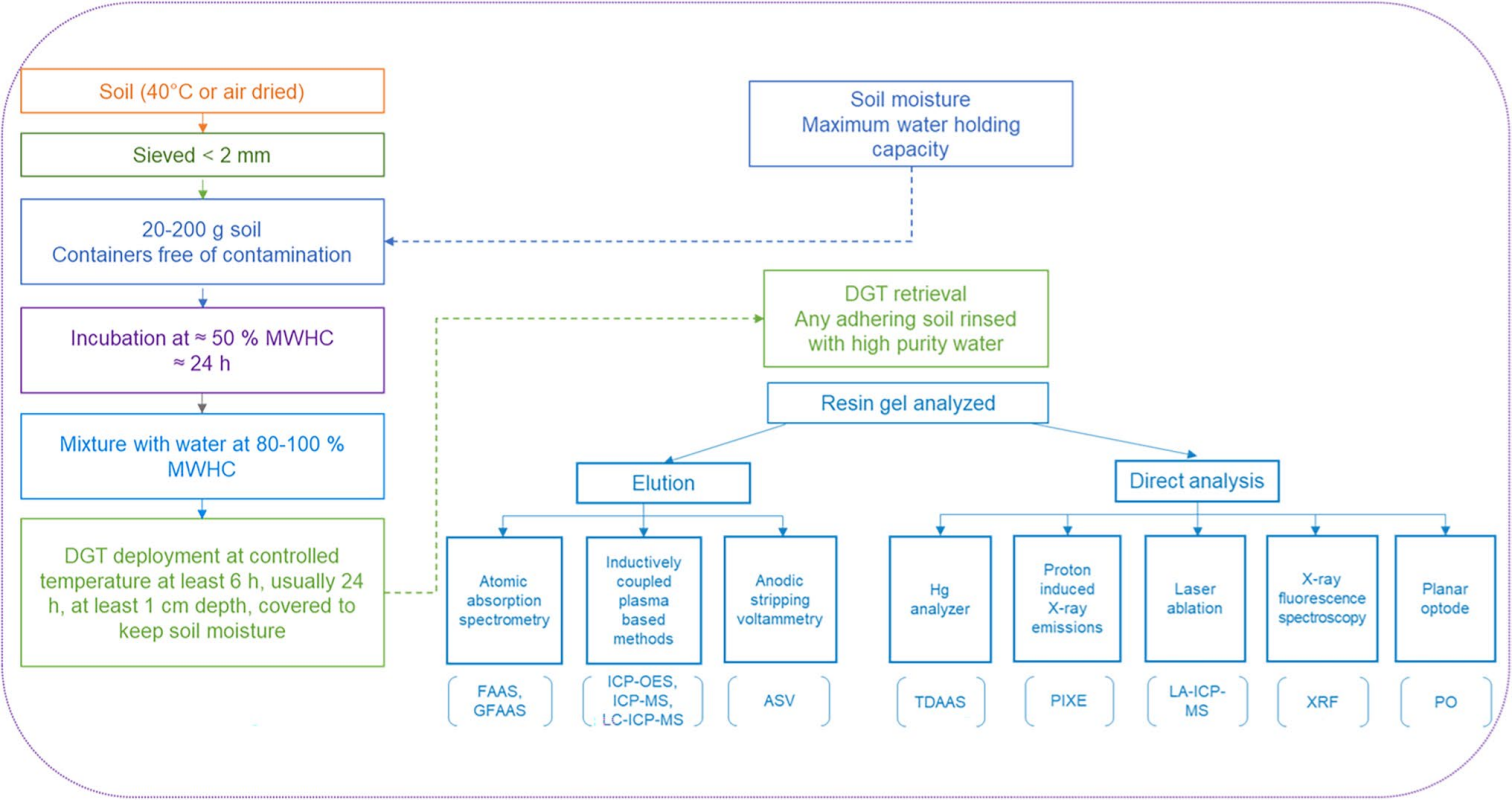
The main steps of DGT procedure for the assessment of DGT-labile fraction of elements in soil

The procedure of using DGT devices into the soil for passive sampling of labile fractions of analytes is based on their deployment saturated with water or at the maximum water holding capacity (MWHC). Other authors used water until 80% of soil MWHC (Babalola and Zhang 2021). The collected soil samples air dried and sieved ≤ 2 mm are mixed with deionized water to MWHC to form a slurry (www.dgtresearch.com). The quantity of soil used is not a very important aspect for typical DGT placement times (24 h). However, a depth of soil adjacent to the DGT sampling device needs to be of at least 1 cm for a deployment period of 24 h considering the depletion of analyte in that area. Typical quantities of soil used per DGT device are of about 20–200 g (Jolley et al. 2016). The soil-water mixture is left for 24 h to equilibrate, while being covered in order to prevent evaporation. It is recommended to perform the DGT deployments under controlled temperature conditions, since this is an important parameter that influences the diffusion coefficients. Deployment periods may vary, but usually they are of about 24 h, which is enough to provide a quantification of the elements (Jolley et al. 2016). A scheme showing the main steps of DGT procedure for the assessment of DGT-labile fraction of elements in soil is presented in Fig. 2.

The analytes retained in the resin gels are usually extracted (using different eluents depending on the analyte types), then analyzed by appropriate techniques, among the most widely used: atomic absorption spectrometry with flame of graphite furnace atomization (FAAS or GFAAS), inductively coupled plasma optical emission spectrometry (ICP-OES), anodic stripping voltammetry (ASV), inductively coupled plasma mass spectrometry (ICP-MS), and hyphenated techniques based on inductively coupled plasma mass spectrometry and liquid chromatography for speciation (LC-ICP-MS). Another possibility is to analyze the gel directly, without a dilution step, using laser ablation ICP-MS (LA-ICP-MS), proton-induced X-ray emissions (PIXE), X-ray fluorescence spectroscopy (XRF) (Wei et al. 2022), or thermal desorption atomic absorption spectrometry (TDAAS)—for Hg analysis (Senila et al. 2023). Also, imaging tools using a DGT gel combined with planar optode (PO) can be used on root systems for high-resolution imaging of solute distribution in porewaters (Santner et al. 2015; Smolders et al. 2020).

### Aspects regarding the DGT use in prediction of nutrient/PTE bioavailability

The bioavailability of a certain chemical substance in soil refers to its freely available fraction, not sorbed or sequestered on soil particles, which is mobile or easily mobilizable, and at which the biota is most exposed (Guan 2019). Meanwhile, bioavailability processes were defined by the National Research Council Committee on Bioavailability of Contaminants in Soils and Sediments as “the individual physical, chemical, and biological interactions that determine the exposure of plants and animals to chemicals associated with soils and sediments” (NRC 2003).

A DGT device deployed the soil slurry represents a sink for analyte labile species in the soil solution. Labile species primarily include the free ion and simple inorganic complexes which dissociate fast enough and have diffusion coefficient values close to that of free ions (Davison and Zhang 2012). Most trace elements, except for Hg, have inorganic complexes that are fully labile within the period of DGT deployment (Letho 2016). Some organic complexes whose sizes are in the colloidal range can also pass through the diffusive gels, but under usual deployment period (hours to days), only limited quantities of colloids pass through the diffusive gels because of their much lower diffusion coefficients (Gao et al. 2019).

When DGT device is deployed in the soil slurry, there may be a considerable depletion of the analyte concentration in the soil solution from the soil adjacent the interface between soil and DGT. To compensate the DGT-induced depletion, elements that are in readily available fraction in the solid phase are re-supplied from the soil solid phase to the solution, contributing to DGT-measured concentration (Degryse et al. 2009; Guan 2019). Consequently, DGT-labile concentration (*C*_*DGT*_) of an element integrates soil properties into only one key parameter (Guan 2019). This aspect is highly important and represents a major advantage of the DGT measurement over the “classical” chemical extraction methods, which do not account the soil properties.

The ratio *R* of *C*_*DGT*_ to the measured concentration of a specific element in soil solution (*c*_*soln*_) is a parameter which displays the soil’s capability to supply solute to the soil solution after the depletion at the DGT-soil interface:

3 $$R=\frac{C_{DGT}}{c_{soln}}$$

For a usual deployment in of DGT devices in soil slurry of 24 h, and a frequently used diffusion layer thickness (0.093 cm), there are three types of solute supply to the DGT (Zhang et al. 1998): (*i*) *sustained case* (*R* > 0.8), which means a continuous and rapid supply of solute from the soil particles to sustain the flux into the DGT; (*ii*) *partially sustained case* (0.2 ≤ *R* ≤ 0.8), or *intermediate case*, when supply of solute forming the soil solid phase exists, but is insufficient to sustain the maximum flux into to DGT; (*iii*) *diffusive case*, or *unstained case* (*R* < 0.2), when the supply for solid phase is extremely low or is no resupply of solutes (Letho 2016).

For the partially sustained case, in which there is a diffusional supply from both the soil solution and release from the solid phase, the interfacial concentration can be associated with the effective concentration of labile species, *C*_*E*_, a concept developed by Zhang et al. (2001). *C*_*E*_ signifies the supply of analyte to any sink, DGT or a crop, that originates from diffusion from two above-mentioned sources. Because only the diffusion concentration is considered, which is a similar process to those occurred in the rhizosphere of plants, *C*_*E*_ can be linked to the uptake of the plant (Marrugo-Madrid et al. 2021). *C*_*E*_ can be calculated as the ratio between *C*_*DGT*_ and *R*_*diff*_, in which *R*_*diff*_ represents the ratio of the time-averaged concentration at the DGT interface to the concentration in the soil solution for diffusion case only.

4 $${C}_E=\frac{C_{DGT}}{R_{diff}}$$

*R*_*diff*_ can be computed using the numerical model 2D-DIFS (two-dimensional DGT induced fluxes in sediments) adapted by Sochaczewski et al. (2007) from the 1D-DIFS software developed by Harper et al. (2000). The DIFS program enables the calculation of the ratio of DGT concentration to total solution concentration, which also depends on the adsorption/desorption kinetics of elements within the soil. The distribution of the labile element fraction between the soil solid and the pore water concentration is measured by the distribution coefficient, *K*_*dl*_ (cm^3^ g^−1^), while for the response time to depletion the parameter *T*_*c*_ (s) is employed (Guan et al. 2017). To calculate *R*_*diff*_ only for the diffusive case, a very large value for *T*_*c*_ or a value for *K*_*dl*_ close to zero should be used. Thus, the DIFS program can be used to simulate *R*_*diff*_, *K*_*dl*_, and *T*_*c*_ (Sochaczewski et al. 2007).

It should be noted that, from the first investigation on DGT as a surrogate for estimating plant uptake of several elements (Cd, Co, Cu, Ni, Pb, and Zn) carried out by Davison et al. (1999) to many other studies reported until present, in general, DGT provided element concentrations better correlated with those accumulated by plants. However, DGT does not always provide the best nutrient/metal bioavailability prediction. This is explained by the fact that bioavailability depends not only on the speciation in soil but also on the receptor (plant characteristics), which DGT cannot account for. For example, plant roots limit toxic metal uptake at toxic concentrations. Thus, the supply from the soil is not totally accumulated, and DGT measurements can overrate metal bioavailability. Also, plant exudates may modify elements’ solubility and mobility in the rhizosphere area (Guan et al. 2022). Thus, as much as possible, the analyzed soil substrate should be carefully chosen in the rhizosphere zone. It should also be considered that the concentration of metals/nutrients in the shoots depends on their own translocation factor (Guan et al. 2022). Other reasons for differences between DGT-predicted bioavailability and accumulation by plants include the higher moisture content in soil during the DGT deployment compared to that during plant growth, which determines higher diffusion fluxes, differences between the period of DGT deployment (usually 24 h) and period of plant growth (weeks), differences between DGT device and roots geometries (that have smaller radius), and presence of root hairs (Degryse et al. 2009; Degryse and Smolders 2016).

DGT has the potential to be deployed in situ, although this direction has not yet been extensively used until now due to potential issues related to the deficiency of parameter control, mainly regarding the soil moisture. Several reports on particular cases of DGT application to wet soils, such as rice fields, were published (Wang et al. 2021; Chen et al. 2022; Wang et al. 2022a).

## DGT applications to nutrient labile fractions in soil

DGT applications to nutrients in agricultural soils deal with studies on phosphorous and selenium bioavailability, but nutrients such as potassium, manganese, and nitrogen forms were also considered in such studies. A total of 26 papers published in recent years have been identified and are discussed in this review. The main trends that can be observed related to the applications of the DGT technique for nutrients in these papers are the evaluation of bioavailability changes after the application of different amendments to soil and the use of DGT for the prediction of the uptake in different crops. The main findings from these researches are detailed below. The effects on P availability were tested after the addition of biochar (Chen et al. 2022; Yang and Lu 2022), or other organic and inorganic fertilizers containing P (Nobile et al. 2018; Kang et al. 2021; Wenzel et al. 2022; Zhang et al. 2023). In all cases, the DGT was reported as a useful tool in assessing the impact of amendments on P availability.

Chen et al. (2022) studied the fluxes of P in the rice rhizosphere using DGT, high-resolution dialysis (HR-Peeper), and zymography techniques. DGT measurements indicated that long-term biochar addition to the soil significantly diminished the diffusion and resupply capacity of P from the soil solid phase to the soil solution, thus lessening the risk of P release into the environment (Chen et al. 2022). Yang and Lu (2022) used a combination of chemical extraction and DGT technique to assess the phosphate availability of in straw/biochar-amended soils, and it was found that biochar augmented P availability and soil pH more than straw returning. Kang et al. (2021) conducted pot and field tests in drip-irrigated calcareous soil and used DGT next to other P analyses to assess changes in its availability in soil. With the aid of the DGT technique, the authors clarified that repetitively releasing P through the fertigation method is suggested as an efficient P application approach in drip-irrigated field. P in soil was also evaluated by the DGT technique and classic extractions in order to control the effects of soil type and fertilizer amendment on the P availability and to assess the ratios of inorganic P vs. organic P in soil (Nobile et al. 2018).

The DGT technique was used to quantify the availability of P in several types of sewage sludge-based fertilizers containing P fertilizers (Vogel et al. 2017). Combinations of fertilizer and soil were incubated for several weeks and DGT devices were immersed at different moments. It was found that plant-available P was obtained after 2 weeks of incubation. In a previous study, Vogel et al. (2021) combined DGT and ^13^P NMR to determine P species in soil. This combination allowed the authors to identify organic P species in solutions. DGT measurements were used to assess the wheat grain yield in long-term fertility experiments (Wenzel et al. 2022). DGT was reported to be superior to the classical quantity and intensity tests, due to its ability to mimic, like plant roots, P diffusion, and resupply from the soil solid fraction. A 5-year fertilization trial was employed to assess the influence on soil P fractionation (Zhang et al. 2023). The P resupply was simulated using DGT and DGT-induced fluxes in soils (DIFS), throughout the maize season, under five conditions of fertilization: no fertilizer, chemical fertilizer, chemical fertilizer joint with bone meal fertilizer, crop straw, and bioorganic fertilizer. With the aid of DGT and DIFS investigation tools, it was observed that organic fertilization, particularly NPKC and NPKM treatments, provided superior enrichment effects on the P supply pool and P resupply for improved plant P uptake.

In other papers, the DGT technique was used to assess P mobility in soils and its bioavailability to various agricultural crops. In general, P uptake by crops or P in soil solution was better correlated with P measured by DGT. P mobility in 75 topsoil samples from a plastic-covered greenhouse vegetable production form China was assessed, and it was found that DGT is a precise predictor of P mobility in soils, since DGT results for P provided a very strong correlation (*r*^2^ = 0.97) with P in soil solution, superior to that obtained by conventional soil extractable P test methods (sodium bicarbonate extractable—Olsen P, ammonium oxalate extractable P, MehlichIII P) (Kalkhajeh et al. 2018). Large-scale experiments were conducted to compare the soil tests for available P in agricultural soil from five countries across Europe (Nawara et al. 2017). P availability was evaluated with five tests: ammonium oxalate, ammonium lactate, Olsen P, CaCl_2_, and DGT, in 11 different soil types, cultivated with different crops. All five tests were positively correlated to the P accumulated by different crops. However, no test was considerably superior than the others, while the oxalate extraction had the weakest correlation. In a later study, Nawara et al. (2018) used the same methods to assess the P bioavailability in agricultural soil, but in depleting P speciation in pot experiments. A strong positive correlation (*r*^2^ = 0.73) was obtained for the P measured by DGT and plant uptake. Tuntrachanida et al. (2022) explored the fractionation and solubility of P in tropical agricultural soils, by combining the DGT technique to measure P fluxes in soil with spectroscopic measurements. With these techniques, the authors were able to find the association between P and different minerals in soil. The DGT concentration of P in the soil solution was extremely variable, with the maximum and the lowermost values detected in acidic and coarse soils, and in alkaline- and fine-textured soils, respectively. The DGT technique and calcium acetate lactate have been used to predict crops (winter wheat and spring barley) response in long-term P fertilization experiments. DGT was found to be superior in predicting P transfer to the two crops (*r*^2^ = 0.42 for barley, *r*^2^ = 0.32 for wheat grain yield, and *r*^2^ = 0.36 for wheat grains) (Hill et al. 2021). The DGT technique has been used to assess the bioavailability of P and other nutrient elements in flooded soils cultivated with rice crop. Wang et al. (2019a) studied, by means of DGT technique combined with high-resolution dialysis, the Fe and P interactions in flooded soils with rice crop. Using this combination of techniques, the authors observed a depletion of P and Fe(II) in porewaters around the rice root area. DGT was also used to evaluate P availability in agricultural soils of Scandinavia (Denmark, Sweden, Norway, and Finland) used in pot experiments for growing spring barley. In this case, DGT was also compared with three frequently used soil extraction methods: sodium-bicarbonate (Olsen P), ammonium lactate (PAL), and ammonium acetate (PAAC) (Mundus et al. 2017). Crops in pot experiments responded to fertilization and accumulated P, but there was a weak or no correlation with extractable P in soil, even with the DGT technique. Since in field conditions, extractable P measured in soils 30 days after establishment predicted P concentrations in leaves of unfertilized plants (*r*^2^ = 0.83), the authors emphasized the significance of pot size relative to the produced biomass in order to avoid the P depletion in areas surrounding the roots.

The DGT technique has also been developed to simultaneously determine the bioavailability of cationic nutrients (e.g., K) and P in agricultural soils by employing new types of retention gels. A resin gel has been created by combining amberlite and ferrihydrite for the simultaneous determinations of two important nutrients in soil, K and P (Zhang et al. 2013). It was showed that the developed gel has the capacity to quantify plant-available P and K in soils. In another study (Zhang et al. 2014), the DGT with the same binding phase was investigated to assess the influence of competing cations in solution on K uptake. The diffusion coefficient for K was lowered by the presence of competing cations. It was also reported that this mixed gel had the capability to quantify Ca and Mg. In a later study, this type of binding gel was used to estimate the necessities for K as fertilizer for wheat in a glasshouse trial (Zhang et al. 2017).

Selenium is an essential micronutrient, of which both deficiency and excess may have negative effect on biota. Thus, the studies of Se bioavailability using DGT in soil rise a high interest for researchers, reflected by eight recent papers dealing with Se bioavailability. DGT with ferrihydrite binding gel were mainly used for Se accumulation, and the results generally well predicted the Se uptake by crops such as *Brassica juncea* from fertilized soils, with correlation coefficients between 0.89 and 0.99 (Dinh et al. 2021), pak choi from soils amended with selenite and selenate, with correlation coefficients of 0.364 and 0.957 for shoots, and 0.909 and 0.876 for roots (Peng et al. 2019), purple cabbage, broccoli, wheat, and mustard from selenite or selenate-amended soils in pot experiments (Peng et al. 2017). Se bioavailability and accumulation in pak choi were evaluated by comparing the DGT technique with the chemical extraction methods (Peng et al. 2020). It was reported that the plant uptake of Se(IV) was better predicted by the DGT technique (*r* = 0.933 for shoots and *r* = 0.943 for roots) than by the chemical extraction methods, while the Se(VI) uptake was also well predicted by DGT (*r* = 0.885 for shoots and *r* = 0.841 for roots), but was better predicted by the KH_2_PO_4_–K_2_HPO_4_ extraction (*r* = 0.913 for shoots and *r* = 0.853 for roots). Wang et al. (2019b) reported on the use of the DGT technique and classical chemical extractions to evaluate the uptake of Se by the maize from naturally enriched soils. The authors found that the DGT can consistently predict the uptake of Se by maize (*r* = 0.933). Zhang et al. (2020) assessed Se bioavailability to *Brassica juncea* in soils, by using DGT and chemical extraction methods. The correlation coefficients between the Se transfer rates to *Brassica juncea* and the bioavailable Se content in soil, measured by different techniques, followed the trend DGT > KCl > water > EDTA> KH_2_PO_4_ > NaHCO_3_ extractions. Sequential chemical extractions and DGT were used to assess Se fractionation in pot experiments. It was found that the Se concentrations were generally resulting from soluble and exchangeable Se fractions (Lyu et al. 2021). However, in another study on soil amended with S and P, the Se content in soil measured by DGT was not significantly correlated with the Se content of pak choi. This may be explained by the fact that DGT cannot reflect the competitive relationship between P, S, and Se at the plant root uptake sites (Jiang et al. 2022).

Kodithuwakku et al. (2023) developed a DGT device for the determination of NO_3_–N and NH_4_–N in soil solutions. The used resin gel was either based on A520E (anion exchange resin) or on PrCH (cation exchange resin), while an agarose-type gel was used as diffusive layer, covered with a polyethersulfone filter membrane. The developed DGTs were able to measure low concentrations of NO_3_–N and NH_4_–N in soils. The concentrations assessed by DGT and by extraction in 2M KCl were significantly correlated for NO_3_–N (*r*^2^ = 0.53).

Table 1 presents selected works observing the use of DGT to assess nutrient mobility in soil and relations with different crops, presenting the analytes, used DGT tools, main experimental conditions, and main findings.

**Table 1:** Selected works observing at the use of DGT to assess nutrient mobility in soil and relations with different crops

Analyte	DGT type	Experimental conditions	Crops	Main findings	References
P	DGT-P/Fe–ZrO-Chelex resin gel to simultaneously measure labile P and Fe; Zr-oxide DGT to measure soil labile P fluxes	DGT was used to in situ assess P availability in rhizosphere, resupply, and release risk to the environment from soil amended with biochar	Rice	Biochar addition to the soil significantly reduced the P availability in rice rhizosphere	Chen et al. (2022)
P	Ferrihydrite hydrogel as binding gel	DGT was used for the assessment of P availability in soil of the Ultuna long-term field experiment	-	P resupply from the solid fraction of soil solution provided a better prediction on the P uptake than the equilibrium-based extractions	Jarosch et al. (2018)
P	0.4-cm-thick precipitated zirconia-based binding gel 0.75 cm polyacrylamide diffusive gel	DGT was used for the assessment of P mobility in 75 topsoil samples from a plastic-covered greenhouse vegetable production form China	-	The DGT accurately predicted the P mobility in acid-neutral and alkaline soils	Kalkhajeh et al. (2018)
P	DGT with a ferrihydrite binding layer	DGT determinations were compared to frequently used chemical soil extraction methods (ammonium lactate, NaHCO_3_, ammonium acetate) and with the transfer rates to crops	Spring barley	All four extraction methods might predict P concentrations in the youngest fully emerged leaf of unfertilized plants, harvested after 30 days of plant growth. DGT soil test showed the best correlation with plant uptake (0.83). For longer growth periods (56 days), the correlation had disappeared.	Mundus et al. (2017)
P	P-DGT form DGT research	DGT was used for the assessment of P mobility and lability in fertilized soil, in pot experiments. Different P application strategies were used	Maize	Different P application strategies affected the soil available P. The C_DGT_-P correlated well with the water-soluble P. The P uptake by the maize plant was better correlated with the C_DGT_-P than the P concentrations in soil, measured by other methods	Kang et al. (2021)
P	P-DGT ferrihydrite-based binding gel	Large-scale study on 218 soil samples from five countries in Europe, analyzed by DGT and other 4 chemical extraction methods; DGT deployment: soil saturation at WHC, 2–48 h	Different crop species	All tests were positively correlated to the P in crops; however, the oxalate extraction was generally poor	Nawara et al. (2017)
P	P-DGT Fe-oxide resin gel and open pore diffusive gel	P available fraction in fertilized soils was assessed by four methods, including DGT; DGT deployment: soil saturation at WHC, 24 h	-	Fertilizer application increased the available inorganic P and favored the available inorganic O relative to the organic P	Nobile et al. (2018)
P	P-DGT ferrihydrite-based binding gel	DGT measurement combined with X-ray absorption near edge structure (XANES) spectra in soil	-	The association between P and different minerals in soil were reported. The DGT concentration of P in the soil solution was highly variable, depending on the soil type	Tuntrachanida et al. (2022)
P and K	DGT with a binging gel containing mixed amberlite and ferrihydrite (MAF)	Elution efficiency was tested by placing DGT with tested gels in solutions with known amounts of analyte for 24 h. The diffusion coefficients were measured by deployment of DGT devices in boxes of 3 L containing known concentration of analyte	-	Elution efficiencies of the resin gel were 90% for K and 96% for P, respectively. The diffusion coefficient of K through the diffusive gel was calculated as 1.30 × 10^−5^ cm^2^ s^−1^ at 22 °C	Zhang et al. (2013)
Se	Ferrihydrite hydrogel as binding gel for Se, and polyacrylamide (0.78 mm thickness) diffusion gel	DGT was used to measure Se bioavailability in soil supplemented with selenite and organic amendment (cow and chicken manures) in pot experiments	*Brassica juncea*	Simultaneous amendment with manure and selenite can result in the reduction of Se mobility in the soil. DGT predicted well the accumulation of Se in *B. juncea*	Dinh et al. (2021)
Se	DGT form DGT research—for Se	DGT was used to measure Se bioavailability in soil amended with P and S in pot experiments	Pak choi	C_DGT_-Se was not significantly correlated with the Se content of pak choi since DGT cannot reflected the competitive relationship between P, S, and Se at the plant root uptake sites	Jiang et al. (2022)
Se	DGT form DGT research—for Se	Pot experiments on soil with Se concentrations varying between 0.12 and 20.62 mg kg^−1^ (average of 2.01 mg kg^−1^). Sequential chemical extractions and DGT were used to assess the Se fractionation in soil	-	Soluble and exchangeable Se fractions with high mobility in soil represent about 0.7% and 5.1% (respectively) of total Se; the DGT-determined concentration of Se was generally resulting from the soluble and exchangeable Se fractions	Lyu et al. (2021)
Se	DGT form DGT research—for Se ferrihydrite hydrogel; polyacrylamide diffusion gel	Soil spiked with Se at a concentration of 1 mg kg^−1^ with selenite and selenate. Soils were aged until 100 days, and analyzed to assess changes in Se availability	Pak choi	DGT may evaluate the fluctuations of Se availability in soil and predict the Se uptake by pak choi crop in aged soils	Peng et al. (2019)
Se	DGT form DGT research—for Se ferrihydrite hydrogel; polyacrylamide diffusion gel	Chemical extractions and DGT technique were used to assess Se content and mobility in soils amended by selenite or selenate	Purple cabbage, broccoli, wheat, and mustard	The soil amended with selenate and cultivated with wheat has a very high C_DGT_-Se compared with soil amended with selenite. Purple cabbage and broccoli changed most obvious change Se mobility with wheat and mustard	Peng et al. (2017)
Se	DGT form DGT research—for Se ferrihydrite hydrogel; polyacrylamide diffusion gel	Soil spiked with selenite and selenite. Chemical extractions and DGT technique were compared and correlated with accumulation by crop	Pak choi	DGT technique better predicted plant uptake of Se(IV) than the chemical extraction methods, while KH_2_PO_4_–K_2_HPO_4_ extraction better predicted the Se(VI) uptake	Peng et al. (2020)
NO_3_–N and NH_4_–N	A520E anion exchange resin and the PrCH cation exchange resin for resin gel Agarose diffusive gels	Experiments assessed the ability of the DGT with A520E anion exchange resin and the PrCH cation exchange resin to measure mobile NO_3_–N and NH_4_–N	-	The mass of NO_3_–N and NH_4_–N accumulated by the DGT device linearly increased on domains of concentrations in soil from 5 to 300 mg kg^−1^ NO_3_–N and from 5 to 300 mg kg^−1^ NH_4_–N; Concentrations measured by DGT and by extraction in 2 M KCl were significantly correlated	Kodithuwakku et al. (2023)

## DGT applications for heavy metal labile fractions in soil

PTEs group includes heavy metals and several metalloids and are considered the main soil contaminants (Bai et al. 2023). Thus, they have received substantial consideration in recent studies. A total of 57 recent publications dealing with DGT in soil focused on heavy metals such as Cd, Pb, Zn, Cu, Cr, etc., while among the toxic metalloids, As was the most studied. In the studies involving metallic cations, the Chelex-type resin gel was predominantly used, while in the metalloids case, the ferrihydrite-type binding gel was mostly used. A particular attention was paid to Hg determination, due to its special characteristics and high toxicity. Numerous studies focused only on this element, probably because the DGT with Chelex binding gel, which is used for a majority of metallic cations, but is not suitable for the Hg determination. Consequently, specialized DGT tools for Hg and its species were developed and used.

### Environmental studies using the DGT for assessing the PTE availability in soil and their transfer rate to crops

DGT has proven to be a beneficial technique for many studies dealing with the availability of PTEs in soil and their transfer to crops, within the 28 papers published in recent years on this topic. PTE measurement by DGT tends to be well correlated with crop uptake. Table 2 displays selected works observing the use of DGT to evaluate heavy metal mobility in soil and their transfer to different crops (maize, wheat, tomato, bean, rice, barley, turnips, eggplants, spinach, potatoes, onion, corn, peppermint, lettuce, garlic, parsley, carrot, pak choi). A tendency that can be observed in these studies is that the DGT is not yet used alone to assess metal bioavailability in soils; other “classical” chemical extractions are used for comparison purposes, demonstrating that DGT still needs to be validated. Another common finding is that in some papers, the DIFS software was used to calculate the element’s effective concentrations. However, other authors preferred to use the *C*_*DGT*_ directly for correlations with crop uptake. While some studies deal with the multi-element determination of PTEs, other studies studied only a specific contaminant, such as Cd, As, or Zn.

**Table 2:** Selected works observing the use of DGT to assess heavy metals mobility in soil and relations with different crops

Analyte	DGT type	Experimental conditions	Crops	Main findings	References
Cr, As, Cu, Zn, Cd, Pb	DGT with Zr-oxide-Chelex binding gel to measure labile metal(oid) concentration in soil	Soil from the rhizosphere of maize and wheat, and from these crops’ tissues were collected and analyzed. DGT, soil solution, EDTA, and total content methods were used to measure metal concentrations	Maize and wheat	Metal contents in soil, measured by DGT, were significantly correlated with their contents in the wheat and maize	Bai et al. (2023)
Zn	DGT with Chelex binding gel; polyacrylamide with agarose diffusive gel	Greenhouse experiment in lysimeters in which different amounts of ZnO nanoparticles were applied. DGT and chemical extractions were used to assess Zn availability	Cherry tomato and common bean	DGT technique was one the most appropriate methodologies for predicting the Zn availability in soil	Almendros et al. (2020)
Pb, Cu, Cd	DGT with Chelex binding gel	Soil samples contaminated at allowable limit with Pb, Cu, Cd, from agricultural soil treated with biochar and *Delonix regia* pods (2% *w*/*w*)	-	Dynamics of metals in contaminated soils is changed after treatment with amendments	Babalola and Zhang (2021)
Cd, Cr, Cu, Pb, Zn	DGT with Chelex binding gel	Industrially contaminated soil samples treated with natural zeolites (3 and 6 wt.%), incubated for 3 months. DGT was used to assess mobility and resupply from the solid phase	-	The resupply of Cd and Pb from the soil solid phase decreased in the samples amended by zeolite	Senila et al. (2022)
Cd	DGT with Chelex binding gel	Single and sequential (BCR) extractions together with DGT were used to predict Zn transfer to maize and release in soil	Maize	Bioavailable Cd concentration in soil assessed by DGT significantly correlated with the Cd accumulated in maize grains	Chen et al. (2021)
Cd	DGT with Chelex binding gel	Single chemical (CaCl_2_, DTPA, NH_4_NO_3_) extractions and DGT were employed to assess Cd bioavailability from amended soil to rice	Rice	The DGT-labile content of Cd in soil was well correlated to Cd content accumulated in brown rice	Luo et al. (2021)
Cd	DGT with binding gel (layered double hydroxide modified by DTPA and DTPA-LDHs)	Single and sequential chemical extractions, DGT, and bioindicator methods were used to assess Cd bioavailability in soils with different aging times	Barley	Cd bioavailability to barley was better evaluated by means of DGT compared with chemical extractions. Soil aging decreases Cd bioavailability	Ma et al. (2022)
Cd	DGT with Chelex binding gel with a dual-mode DGT holder, jointly with an agarose diffusive gel	Soils and vegetables sampled from greenhouse vegetable production systems. Total, CaCl_2_ extractable, and DGT-labile Cd concentrations were measured	Turnips and eggplants	DGT labile concentrations are independent of soil properties and may offer the possibility to obtain new soil thresholds in future research	Tian et al. (2018)
Cd	DGT with Chelex binding gel and polyacrylamide diffusive gel	Three water-management treatments (continuous flooding, intermittent flooding, and non-flooding) were compared to observe their effects on Cd phytoavailaility. DGT technique was utilized to display soil labile Cd and Fe	Rice	The Cd phytoavailability were generally in the order of non-flooding > intermittent flooding > continuous flooding. The Cd amount in roots experienced the same trend as DGT-Cd	Wang et al. (2020)
Cd	DGT with Chelex binding gel and agarose cross-linked polyacrylamide diffusive gel	Corresponding soil and plant samples were collected from different farms. Cd concentrations were measured in the plants, while in the soil four tests were applied to investigate the bioavailability of Cd: pseudo total, porewater, Ca(NO_3_)_2_-extracted, and DGT Cd concentrations	Spinach, potatoes, onion, wheat	Cd concentrations in soil measured by the different extractions were positively correlated to accumulation in crops. The extraction in Ca(NO_3_)_2_ well predicted Cd transfer in spinach leaves and onion, while DGT and porewater Cd better estimated the Cd transfer to potatoes and wheat grains	Yi et al. (2020)
As, Pb, Cd, Ni, Cu, Cr, Mn, Zn, Ba, U, REEs	DGT with Chelex binding gel	Multi-method evaluation of elements’ concentrations/fractionation/speciation in soils, based on sequential extraction (BCR), single extraction, and DGT	Corn	All techniques for available fraction assessment provided results well correlated to the metals accumulated in the corn plant, as compared to total metal contents.	Galhardi et al. (2020)
Cd, Cu, Pb, Zn	DGT with Chelex binding gel	DGT combined with BCR sequential extraction and bioaccessibility methods to estimate potential bioavailability and release of metals	-	Significant positive correlations obtained between the DGT and the bioaccessible fraction of metals	Li et al. (2023)
As, Cu, Zn	DGT with Chelex and with ferrihydrite binding layers	DGT was compared with other two analytical approaches (macrocolumn and microcolumn tests) to assess the changes in availability of Cu, Zn, and As in soil treated with different amendments	-	Microcolumn test was the fastest testing instrument to reveal the efficiency of amendments, while DGT is a good alternative to this, having less technical demands.	Manzano et al. (2019)
Cd, Pb, Zn	DGT with acrylamide monomer and allylagarose cross-linker as diffusive layer	DGT with a new type of diffusive gel was used to measure metal bioavailability in contaminated soil treated with sewage sludge under peppermint cultivation conditions	Peppermint	DGT compiled with 15% acrylamide and 0.3% allylagarose can substitute the original gel cross-linked with agarose to assess Cd, Pb, Zn mobility	Mohseni et al. (2021)
Zn	DGT with Chelex binding gel, agarose diffusive gel	Greenhouse experiment on contaminated soil treated with sorghum, poultry manure, and clover residues to assess changes in Zn bioavailable fraction using DGT, DTPA extraction, and bioassay	Corn	Application of organic fertilizers increases Zn effective concentration (measured by DGT), Zn extracted in DTPA, and plant Zn concentration. Sorghum residues decreased Zn effective concentration.	Mohseni et al. (2022)
As, P	DGT with cerium oxide resin gel	CeO_2_-DGT were deployed in four types of farmland soils over 400 h to study the interactions between P and As during their migration behaviors. These were coupled with dynamic DGT induced flux model in soils	-	The collected quantities of As in the DGT tools proved negative correlation with those of P, suggesting their competitive adsorption on soil solids	Ren et al. (2022)
Cd, Cr, Cu, Pb	DGT with Chelex binding gel	Greenhouse experiment on contaminated soil amended with biochar and compost, cultivated with lettuce. BCR sequential extraction, pore water extraction, and DGT were used to assess bioavailability of Cd, Cr, Cu and Pb and the effect of amendments on this	Lettuce	The amendment of soil with compost and biochar significantly decreases metal uptake by lettuce. DGT and the sequential extraction procedure results were better correlated with plant uptake than those from the pore water extraction and from total metal measurement	Turull et al. (2021)
Hg	DGT with open (polyacrylamide crosslinked with agarose) gel and restricted diffusive (polyacrylamide crosslinked with bis-acrylamide) gel layers; 3-mercaptopropyl-functionalized silica gel binding layer	Open and restricted diffusive gels to distinguish between inorganic and organic bioavailable Hg species in soil. The effective Hg concentration in soil was evaluated for predicting its uptake by lettuce in pot experiments	Lettuce	Linear relationships between the mass of Hg gathered in the resin gels and Hg concentration in the solution were obtained for both open and restricted gels. Hg concentrations in soil measured by DGT fitted well to the Hg concentration measured in lettuce roots.	Turull et al. (2019a)
Hg	DGT with open gel and restricted diffusive gel layers; 3-mercaptopropyl-functionalized silica gel binding layer	Greenhouse experiment on contaminated soil, amended with biochar and compost, and cultivated with lettuce. DGT with open gel and restricted diffusive gel used to measure organic and inorganic species	Lettuce	Added biochar lowers the bioavailability of Hg in soil and thus reduces the Hg uptake by lettuce. Inorganic Hg were the most abundant Hg species in soils, in all sets of the experiment	Turull et al. (2019b)
Hg	DGT with 3-mercaptopropyl-functionalized silica gel binding layer	Four species of vegetables and corresponding garden soils collected from four locations around a former mining area. Total Hg in soil and in vegetables, Hg in soil solution, and DGT-labile Hg fraction in soil were measured	Garlic, parsley, carrot, onion	The R ratio between Hg-DGT/Hgsoln (on average 0.75) suggested a high resupply probability of Hg from soil solid phase to the soil solution	Senila et al. (2023)

Bai et al. (2023) correlated directly *C*_*DGT*_ of PTEs (Cr, As, Cu, Zn, Cd, and Pb) from rhizosphere soil with the accumulation by maize and wheat. For comparison, the rhizosphere soil samples were analyzed for their PTE total content and extractible forms in soil solution and EDTA and DGT-labile fractions. Among the methods used in soil analysis, DGT was reported to provide, in general, more accurate results in simultaneously predicting PTE transfer to crops, especially for grains. Also, DGT bioavailability prediction was less impacted by soil pH than other extractions.

Galhardi et al. (2020) used sequential extraction (BCR), single extraction, and *C*_*DGT*_ to assess the fractionation of As, Pb, Cd, Ni, Cu, Cr, Mn, Zn, Ba, U, REEs (La to Lu). The authors used DGT with Chelex resin gel to assess all available fractions of PTEs and REEs. They reported that chemical extractions and DGT correlated better with the metal bioavailability to corn, as compared to the total metal contents in soil. *C*_*DGT*_ of PTE (Cd, Pb, Cu, Zn, Co, Cr, Mn, Ni, and Fe) and PTE concentration in soil solution were measured (Senila et al. 2024). A similar trend of soil resupply capacity (estimated by the R-ratio between the *C*_*DGT*_ and the concentration in the soil solution) was observed with the bioaccumulation factors in *Russula virescens* mushroom. *C*_*DGT*_ was also measured for Fe, Mn, Cd, and As labile fractions in flooded paddy soils (Wang et al. 2022b) or to study the effect of soil redox modifications on the activities of Cd and Cu in soil (Wang et al. 2023a). A new type of resin gel combined with layered double hydroxide nanoparticles changed with diethylenetriaminepentaacetic acid was created for the use of DGT tools to measure eight anions and cations in waters and soils (Wang et al. 2023b). A hyperaccumulator plant of Cd and Zn, *Sedum plumbizincicola*, was grown in a pot experiment (Zhou et al. 2019). Soil total Zn and water soluble, CaCl_2_-extractable, and *C*_*DGT*_ Zn concentrations were measured and used to predict the shoot Zn concentration. Dissimilar to many other studies, in this study, CaCl_2_ extraction produced the strongest correlations with plant uptake than *C*_*DGT*_, results that were explained by two main reasons: Zn uptake *by S. plumbizincicola* was not limited to diffusion, while the behavior of hyperaccumulating species to increase metals solubility by the release of organic acids, which increase metal uptake. DIFS model was used to assess *C*_*E*_ for As, Cr, Cu, Pb, and V in urban soil (Xu et al. 2019a). Cd bioavailability in soil was extensively studied both by *C*_*DGT*_ as well as by estimating its *C*_*E*_ using DIFS. Thus, DGT and DIFS were employed to evaluate the Cd bioavailable fraction and to predict its transfer to maize and its mobility in agricultural soils (Chen et al. 2021). Bioavailable Cd measured by DGT was significantly correlated with Cd accumulated in maize grains (*r*^2^ = 0.92).

*C*_*DGT*_ also was found to predict well Cd transfer to crops. Thus, *C*_*DGT*_ in agricultural soils was reported to be positively correlated (*r*^2^ = 0.95) with Cd uptake by pak choi in a greenhouse experiment (Dai et al. 2017), by cocoa bean (*r*^2^ = 0.5) (Gramlich et al. 2018), and turnips and eggplants grown in greenhouse vegetable production systems (*r*^2^ values from 0.53 to 0.70) (Tian et al. 2018). *C*_*DGT*_ Cd was used to predict Cd uptake by the hyperaccumulator plant *S. plumbizincicola* in different agricultural soil categories. Using *C*_*DGT*_ and piecewise equations, Cd uptake could be predicted at different intervals of soil properties (Wu et al. 2018). In a study on Cd uptake from agricultural soil to spinach leaves, potato tubers, onion bulbs, and wheat grain grown across New Zealand (Yi et al. 2020), the extraction in Ca(NO_3_)_2_ predicted more than 76% of the variability in the Cd concentrations in onion bulbs and spinach leaves, whereas *C*_*DGT*_ and porewater Cd concentration better estimated the Cd transfer to potatoes and wheat grains.

*C*_*DGT*_ in rhizosphere soil of several crops (rice, corn, peanut, and sweet potato) also showed good correlation with its accumulation in those crops (*r*^2^ in the range 0.64–0.90) (Guo et al. 2023). In a study on paddy soil, *C*_*DGT*_ of Cd was correlated with Cd concentration in the rice grains, straws, or roots. *C*_*DGT*_ showed a significant correlation with Cd in crop parts (*r* = 0.733 for grains, *r* = 0.833 for straw, and *r* = 0.680 for roots) (Li et al. 2018). In another study, a significant correlation (*r* = 0.818) was obtained between *C*_*DGT*_ Cd in paddy soil and Cd in rice grains (Xiao et al. 2020). Soil chemical extraction and soil-plant transfer modeling methodologies were used to predict the Cd bioavailability to rice in a large-scale experiment on samples collected from 278 sites in the Guangxi province, China (Wen et al. 2020). *C*_*DGT*_ Cd well estimated Cd in rice grains (*r*^2^ = 0.73), better than Cd in soil solution (*r*^2^ = 0.43).

Almendros et al. (2020) used the *C*_*DGT*_ and low-molecular-weight organic acids (LMWOAs), CaCl_2_, DTPA-TEA, water, and NH_4_Ac chemical extractants to predict the Zn transfer to crops (tomato) from soils amended with ZnO nanoparticles in a greenhouse experiment. The Pearson correlation coefficients (*r*) between log-transformed values of Zn concentrations in crop and *C*_*DGT*_ ranged between 0.787 and 0.915. Later, the same group of research reported *C*_*DGT*_ for Zn well correlated with Zn accumulated by beetroot and green pea, with Pearson correlation coefficients between log-transformed values of the Zn concentrations in crops and *C*_*DGT*_ in the range 0.78–0.96 (Almendros et al. 2022).

Another element in soil extensively studied by DGT was As. Ren et al. (2022) studied the absorption/desorption of As in farmland soils, together with soil P. Cerium oxide–based DGTs were used for the simultaneous determination of As and P in four different categories of agricultural soils in order to investigate As and P migration behaviors. It was reported that both P and As can attain the equilibrium of resupply in 0.7–18 min under DGT depletion. The lability of As and Sb in agricultural conditions in anthropogenic contaminated soils was assessed using various approaches: *C*_*DGT*_, soil solution extraction, and sequential extraction and accumulation by radish As and Sb contents in radish were significantly correlated (*r*^2^ = 0.97–0.99) with their accumulation in crop tissues (Ngo et al. 2016; Ngo et al. 2020). DGT combined with the high-resolution dialysis (HR-Peeper) technique was employed to obtain the distributions of soluble Fe(II), soluble reactive P, and labile P and Fe in the root area of rice (Wang et al. 2019a). DGT with 2.0-mm vertical resolution and hydrochemical monitoring were used to investigate the effects of flooding and flora on the Fe-redox and hydrochemical change in the soil porewater (Wu et al. 2021). It was found that the release of Fe(II) from the wetland rhizosphere due to flooding could have an effect on the release of Fe-associated metals from the riparian marshland to the surface water.

### Studies using DGT as tool for the evaluation of the PTE mobility changes in soil remediation processes

The DGT technique has been used to assess the PTE mobility variations in soil remediation processes in 24 recent papers examined in this review. Among the studied topics, the effects on PTE mobility in soils were tested after the addition of organic amendments (Grüter et al. 2019; Luo et al. 2021; Mohseni et al. 2021; Sun et al. 2022; Zhao et al. 2022), biochar (Bidar et al. 2019; Babalola and Zhang 2021; Gao et al. 2022a, b), and inorganic amendments and sludge (Neu et al. 2018; Xu et al. 2019b; Manzano et al. 2019; Zhang et al. 2019; Mohseni et al. 2021; Senila et al. 2022; Zhang et al. 2022a; Zhang et al. 2022b). Other authors used DGT to assess the effect of soil moisture in PTE mobility (Li et al. 2015; Wang et al. 2020; Zhao et al. 2021; Wang et al. 2022b) or to evaluate the effect of hyperaccumulator plants in PTE mobilization in rhizosphere (Senila et al. 2013; Li et al. 2016; Zheng et al. 2019). Also, the effect of aging periods on PTE mobility in soil was evaluated by DGT (Ma et al. 2022).

Organic amendments, as determined by DGT and other chemical extraction of uptake by crops, generally decreased the PTE mobility in soils. Thus, long-term fertilization with organic matter was found to reduce the Cd transfer in wheat. However, the Zn transfer was not reduced (Grüter et al. 2019). Luo et al. (2021) reported that five of the soil amendments used in their research reduced the transfer of Cd to rice, and that the DGT estimated with precision the Cd bioavailable to rice. In another study, the Zn contaminated soil was treated with sorghum, poultry manure, and clover residues (Mohseni et al. 2021). Generally, due to an increasing dissolved organic carbon and a decreasing soil pH, the bioavailability increased in the amended soil. However, sorghum residues reduced the phytotoxicity risk of Zn. Sun et al. (2022) studied the stabilization of Zn in agricultural soil after the application of organic fertilizer and zeolite. The authors used the DGT, DTPA extraction, and accumulation in Chinese cabbage biomass to assess the reduction of Zn mobility in soil after the treatments. Zhao et al. (2022) employed the DGT to monitor the Cd mobility in soils interacted with controlled-release fertilizers coated with microplastics.

Biochar, a biomaterial produced during the pyrolysis of biomass, was also applied as soil amendment in which the DGT was employed to assess the PTE mobility changes. Thus, Babalola and Zhang (2021) used DGT to evaluate the available concentrations of Pb, Cu, and Cd in soils treated with biochar and *Delonix regia* pod biomass. Biochar modified with magnetite nanoparticles was reported to reduce Cd bioavailability to rice (Gao et al. 2022a, b). The DGT study indicates that biochar modified with magnetite nanoparticles may decrease the replenish capacity of soils to soil pore waters and thus limit the crop uptake. Bidar et al. (2019) studied the immobilization of Cd, Cu, Pb, and Zn in contaminated brownfield and agricultural soils treated with wood biochar and iron grit.

Other studies were focused on the effects of inorganic amendments and sludge application on soil in PTE mobility. DGT was compared with other two analytical approaches to measure the possible changes in the availability of Cu, Zn, and As in polluted soils, after a co-application of paper sludge alkaline waste and iron sulfate (Manzano et al. 2019). The DGT was found to be a good alternative to assess metal mobility, with less technical requirements. Using DGT to measure Cd, Pb, and Zn bioavailability in soil treated with sludge, Mohseni et al. (2021) estimated that the sewage sludge treatment rises the resupply of these elements to soil solution and the effective concentration of Cd, Pb, and Zn. Senila et al. (2022) examined the influence of natural zeolite amendment to contaminated soil on heavy metal (Cd, Cr, Cu, Pb, and Zn) availability over a 3-month period of incubation. A decrease of Cd and Pb mobility in the soil solid phase from the samples treated with zeolite was observed. The immobilization of metals (Cd, Pb, Zn) and As in agricultural soils contaminated with drinking water treatment residues was tested in pot experiments (Neu et al. 2018) and assessed by DGT. Xu et al. (2019b) studied the effect of phosphate application to the agricultural soil on the availability of Pb. Using DGT, in situ solution extraction, and EDTA extraction methods, a mobilization effect of the amendment application on Pb in soil was observed. Zhang et al. (2019) reported the use of a sequential extraction procedure coupled with the DGT technique to assess the influence of the ferrihydrite dissolution/transformation process on the availability of As in soils. In another research, As was extracted from polluted paddy soils with ferrihydrite-loaded sand columns (Zhang et al. 2022a). The process was investigated using mesocosm coupled with DGT for in situ visualization. Zhang et al. (2022b) used the DGT to assess the effects of soil remediation by nanoscale zero-valent iron on heavy metal (Cr, Cu, Zn, Pb) bioavailability in soil.

The content of water in soil along with the way it is managed also has a role in PTE mobility. In a study, DGT was used to examine the effects of the soil-drying processes on metal availability in contaminated soil. Soil moisture influences the metal availability, but it is dependent on the metal species and soil types (Li et al. 2015). Three water-management treatments, namely continuous flooding, intermittent flooding, and non-flooding, were conducted in pot experiments to assess their effects on the Cd phytoavailaility in three types of paddy soils and the Cd accumulation in rice (Wang et al. 2020). The DGT technique measured the available Cd in soil and provided the most trustworthy prediction for the Cd accumulation in rice. Zhao et al. (2021) studied the effect of sulfur on soil Cd mobility under flooded conditions in the soil-rice system with the aid of the DGT technique. It was found that the synergistic effect of Fe and S reduced the mobility of Cd. Wang et al. (2022b) used DGT and soil pore water sampling to explore the impacts of various types of S application on the bioavailability of Cd. It was reported that soluble and labile Cd concentration was immediately fixed in soil after flooding, but activated next to the rice transplantation.

Hyperaccumulator plants can modify the PTE mobility in rhizosphere by their roots. The effects of phytoextraction using the hyperaccumulator *S. plumbizincicola* on Cd and Zn availability, desorption kinetics, and speciation in contaminated soils were examined by chemical extraction and by the DIFS model (Li et al. 2016). The effects of two fern species on the mobility changes in rhizosphere soil were investigated in pot experiments using DGT (Senila et al. 2013). An increase of As labile fraction was observed in the rhizosphere of the fern species, a hyperaccumulator, in the grown plant. Zheng et al. (2019) studied the influence of biochar addition on the phytoextraction process of an As hyperaccumulator. A pot experiment was conducted to investigate the biochar effect on the As transfer in *Pteris vittata* fern. The DGT technique was utilized to characterize the migration of As in soil. The results showed that phytoextraction meaningly decreases Cd and Zn availability and that the hyperaccumulator has a significant role in the mobilization of less available metal fractions by repeated phytoextraction.

The effect of different aging times on Cd bioavailability in soil was evaluated by means of chemical extractions, DGT technique, and biological indicators (toxicity to barley) (Ma et al. 2022). It was observed that aging decreases the Cd bioavailability and transfer to barley. The plant root is more appropriate for predicting the Cd transfer from soil, while the plant shoot can well assess the toxic effect of Cd stress on plants. A better evaluation of the Cd bioavailability to barley was reported for the DGT, compared to the classical chemical extractions.

### Studies using DGT as tool for the evaluation of Hg bioavailability in soil

Hg exhibits several features which differentiate its assessment in soil from other metals. Five recent studies on Hg in soil measured by DGT were examined in this review. In the case of Hg, the specific resin gels used in the DGT units contain thiol groups with a high affinity for Hg, such as spheron-thiol or 3-mercaptopropyl-functionalized silica gel (Turull et al. 2019a). In order to differentiate the inorganic and organic labile Hg species in soil, Turull et al. (2019a) used open and restricted diffusive gels. Polyacrylamide crosslinked with agarose was used as open pore diffusive gel, while, as restricted pore layer, a polyacrylamide crosslinked with bis-acrylamide gel was prepared. Both open and restricted gels provided linear relationships between the mass of Hg collected in the resin gels and the Hg concentration in the solution. The two types of DGT units were successfully used to measure inorganic and organic Hg species in soil and were confirmed to effectively predict the Hg uptake by lettuce plants. The DGT technique was also used to measure the bioavailability of Hg in agricultural soils amended with organic fertilizers (biochar and compost), and predicted its uptake by lettuce (Turull et al. 2019b). Both open and restricted diffusive layers were also used in this study to test organic and inorganic Hg species in soils.

The DGT and the DIFS instruments were implemented to examine the Hg resupply kinetics, diffusion, and availability in a paddy soil exposed to flood-drain-reflood management and straw application (Yang et al. 2023). The straw amendment restricted the bioavailability of Hg in the porewater by lessening its resupply capacity, while the transformation into MeHg was substantially enhanced after straw application. Pelcová et al. (2019) studied the mercury bioavailability to *Pisum sativum L*. in soils and compared the bioaccumulation with the DGT measurements. Significantly positive correlations were reported between the Hg-DGT flux and the Hg flux into the plant root, leaf, and stem. The DGT total and labile Hg concentrations in garden soils from a former nonferrous metal mining area and the transfer to crops were evaluated (Senila et al. 2023). It was found that, on average, 84% of the Hg content in soil solution was found in its DGT-labile form.

## Conclusions and perspectives

The DGT technique relates the diffusive flux of analyte to the gradient of the concentration. The current review provides an update of the existing DGT applications in the agricultural field, mainly for assessing the nutrients’ and PTEs’ labile fractions and bioavailability in soil. Recent DGT research focus not only on the nutrients’ bioavailability in agricultural soils, mostly P, Se, K, Mn, and nitrogen compounds, but also on the soil and plant contamination with potentially toxic elements, such as Hg, Cd, Pb, Zn, Cu, Cr, and As. Most of the reviewed literature reported significant positive correlations of the DGT labile fraction with the concentration in soil solution and with the bioaccumulated concentrations of the studied analytes in various biological indicators. In order to obtain accurate results, specific binding and diffusive layers were developed for certain analytes, such as Hg, Cd, Zn, P, and Se, although, in most cases, Chelex-based binding gels for cations and ferrihydrite-based hydrogels for oxyanions were mainly used. Generally, the DGT technique was more predictive for the soil mobility and bioaccumulation rates of the analytes than the classical extraction and fractionation methods, while being more compliant to the safety and environmental requirements.

Currently, studies on DGT deployments *in situ* are limited mainly to flooded soils. However, the development of DGT for in situ deployment for non-flooded soils is particularly interesting. Clear procedures for controlling soil parameters during the DGT deployments should be established for this. Another possible future development is the production of tools capable of expanding the number of target analytes that can be simultaneously determined.

Because DGT has several limitations in mimicking some key processes in dynamic environments, future research to link DGT results with bioassays and equilibrium-based methods will offer a more complete understanding of element bioavailability. Although DGT is a very promising technique and has several unique features which offers information on element bioavailability and toxicity that can be integrated to improve the regulatory work in toxicological and environmental fields, extensive research to produce larger datasets is still necessary (Guan 2019).

DGT as a passive sampling tool is already well integrated in the general tendency in analytical chemistry to achieve greener methodologies. It can improve the limits of quantification, eliminate the interferences by separation of analytes from the complex matrices, thus contributing to the improvement of the performances of analytical methods, reduces the number of necessary steps of analytical procedures, can replace or eliminate the use of toxic reagents in sample preparation, and can be used for performing multi-parameter analysis. Since different binding gels must still be used for the analysis of different nutrients/PTEs, the production of gels capable of expanding the number of target analytes simultaneously determined can be a future development.

## Data Availability

The relevant data from this research are available in the authors’ repositories.
